# Heart Transplantation in Biventricular Congenital Heart Disease: Indications, Techniques, and Outcomes

**DOI:** 10.2174/157340311797484196

**Published:** 2011-05

**Authors:** Bassem N Mora, Charles B Huddleston

**Affiliations:** Section of Pediatric Cardiothoracic Surgery, St. Louis Children’s Hospital, Washington University School of Medicine, St. Louis, Missouri, United States of America

**Keywords:** Perioperative and long-term outcomes, Biventricular, Cardiac transplantation, Congenital heart disease, Failing ventricle, Heart failure, Heart transplant, Orthotopic heart transplantation, Pediatric cardiac surgery.

## Abstract

Heart transplantation is an accepted therapeutic modality for end-stage congenital heart disease for both biventricular and univentricular anomalies. Many transplant centers have pushed the limits of transplantation to include patients with high pulmonary vascular resistance, high panel reactive antibodies, positive cross-matches, and ABO-incompatibility. Excellent results have been possible, particularly with the development of improved diagnostic and therapeutic algorithms to prevent and treat rejection, infection, and post-transplant lymphoproliferative disease. Late graft failure and chronic rejection remain vexing problems. The vast majority of patients with biventricular congenital heart disease have undergone prior cardiac surgical procedures. Indications for transplantation in this subgroup are primarily progressive refractory heart failure following prior cardiac surgical reconstructive procedures. Contraindications to transplantation mimic those for other forms of end-stage heart disease. A determination of pulmonary vascular resistance is important in listing patients with biventricular congenital heart disease for heart transplantation. Modifications in the implant technique are necessary and vary depending on underlying recipient anatomy. Risk factors for perioperative outcomes in patients with biventricular congenital heart disease include the need for reoperation, the degree of anatomic reconstruction necessary during the implant procedure, and the degree of antibody sensitization, in addition to a number of other recipient and donor factors. Postoperative outcomes and survival are very good but remain inferior to those with cardiomyopathy in most series. In conclusion, patients with end-stage biventricular congenital heart disease represent a complex group of patients for heart transplantation, and require careful evaluation and management to ensure optimal outcomes.

## INTRODUCTION

The first pediatric heart transplant was performed in 1967 for an eighteen day old patient with congenital heart disease (CHD) consisting of Ebstein’s anomaly [1]. The allograft survived six hours. It was not until twenty years later, following the introduction of cyclosporine A, that the next pediatric heart transplant was performed. In the 1980’s and 1990’s, heart transplantation became an accepted treatment modality for end-stage heart disease for adults and children. Due to the shortage of donor organs, mechanical circulatory support devices have been used with increasing frequency in the management of end-stage heart disease, particularly in adults. In the past five years, the Berlin Heart ventricular assist device has been used with greater frequency in children and neonates, although this device is not formally approved for widespread use in the United States. To obviate the donor shortage and the prolonged waiting time in pediatric heart transplantation, other recent advances have included transplantation across a positive cross-match and ABO-incompatible transplantation in selected patients up to two years of age.

There is an increasing body of literature concerning heart transplantation for CHD, both in pediatric and adult recipients. Since the majority of CHD lesions can be repaired or palliated, primary heart transplantation has a very limited role in the management of these patients. This is in contradistinction to the 1980’s, when a number of centers, including ours, performed primary heart transplantation for hypoplastic left heart syndrome (HLHS). Improved outcomes following staged palliation for single-ventricle lesions, including HLHS, as well as inevitable waiting list mortality, have swung the pendulum towards palliation or repair of CHD rather than primary transplantation. As a result, in the last two decades, the majority of CHD patients undergoing heart transplantation have failed prior cardiac surgical procedures. 

There is little data regarding heart transplantation for CHD patients with only biventricular physiology. Published studies have grouped all patients with CHD into one entity, and compared the outcomes of CHD patients to other patient populations, most notably cardiomyopathy. The largest single subset of patients with CHD who undergo heart transplantation have failed surgical palliation for single-ventricle lesions. End-stage CHD of the biventricular form is a heterogeneous group of patients, almost all of whom have undergone prior surgical repair. This monograph will highlight the indications, contraindications, recipient anatomic considerations, donor evaluation, surgical techniques, survival, and long-term outcomes following heart transplantation for biventricular CHD, both in adult and pediatric recipients. Since there are no randomized clinical trials, the recommendations are based on single-center or registry reviews as well as expert clinical opinion. Further, since results for patients with biventricular versus univentricular CHD have not been routinely compared in most studies, overall conclusions about survival and outcomes for patients with only biventricular CHD are difficult to determine, particularly considering the wide heterogeneity in recipient diagnoses, prior operations, age, and clinical status.

## INDICATIONS

The indications for heart transplantation are covered elsewhere in this issue. There are three broad subsets of patients: those with CHD, those with cardiomyopathy, and those undergoing retransplantation. Since 1990, the percentage of patients with CHD has been decreasing while the percentage of patients in the other two categories has been increasing. This is primarily due to the avoidance of heart transplantation as primary therapy for patients with HLHS. In the current era, CHD is present in two thirds of pediatric recipients, compared to 80% in earlier eras when transplantation was used as primary therapy for HLHS [2]. Data from the 2010 International Society for Heart and Lung Transplantation Registry indicate that CHD is present in 63% of patients < 1 year of age, 37% of patients 1-10 years of age, 25% in patients 11-17 years of age, and 2% of adult recipients [3]. The diagnosis of CHD became less prevalent across all age groups over the past decade. This was most pronounced in infants. On the other hand, cardiomyopathy and retransplantation have been increasing in frequency as indications for heart transplantation.

In biventricular CHD, there are three main indications for heart transplantation:

- **Refractory heart failure:** this represents the most common indication for heart transplantation. While the long-term results of surgical repair for CHD are quite good, survival is inferior to that of the general population. A number of patients will have poor systemic ventricular function, often late following palliation or correction of CHD. This may be due to myocardial injury sustained during repair, poor myocardial preservation during surgery, or idiopathic progressive ventricular dysfunction, all of which are amenable to heart transplantation. While any patient with CHD can experience heart failure, the more common diagnoses are listed below:
  - **Transposition of the great arteries (TGA):** Patients with D-TGA who underwent the Mustard or Senning atrial switch procedures in the 1970’s and 1980’s can experience progressive heart failure since the systemic ventricle is a right ventricle. This can result in progressive ventricular dysfunction, resulting in severe systemic atrioventricular valve insufficiency. Survival is inferior to age-matched controls [4]. Another group of patients are those with L-TGA who underwent classical surgical repair whereby the systemic ventricle remains a right ventricle. Although sudden cardiac death may occur, most deaths are due to progressive systemic right ventricular failure, which is treatable with cardiac transplantation.
  - **Tetralogy of Fallot:** Patients entering the third decade of life following repair of tetralogy of Fallot have decreased survival compared to age-matched controls [5]. In some, mortality is from sudden cardiac death thought to be due to ventricular tachyarrhythmias. In others, there is progressive heart failure, which would be amenable to heart transplantation.
- **Unreconstructable CHD:** with advancements in cardiac surgical techniques, this entity has become increasingly rare. With the plateau of pediatric heart donors in the United States at 360 annually, there is significant waiting list mortality, particularly for the youngest patients. As a result, every attempt is made to repair or palliate CHD, in order to prolong the use of the native heart as long as possible. A number of rare lesions not amenable to surgical intervention are listed below, most of which fall in the category of univentricular CHD:
  - **Coronary circulation anomalies: **Patients with pulmonary atresia with intact ventricular septum (PAIVS) along with right-ventricle dependent coronary circulation are at high risk for sudden death due to tenuous coronary perfusion [6]. This is usually in the spectrum of univentricular CHD. Likewise, patients with HLHS of the mitral stenosis and aortic atresia variety can have coronary sinusoids from the small hypertensive left ventricle. In a subset of these patients, the native coronary arteries may contain stenoses, which would make surgical palliation less attractive. Cardiac catheterization should be performed in these two patient groups: those with native coronary artery stenoses should undergo consideration for primary heart transplantation as the mortality of surgical palliation is high.
  - **Neonatal Ebstein’s anomaly:** Patients with severe neonatal Ebstein’s anomaly have marked cardiomegaly from severe tricuspid insufficiency, significant right atrial dilation, poor right ventricular function, and functional pulmonary atresia, with poor antegrade flow into the pulmonary arteries. Surgical repair carries high risk in symptomatic neonates with severe Ebstein’s anomaly [7]. Primary heart transplantation should be considered on a case-by-case basis.
  - **Severe multi-valve disease:** Most atrioventricular or semilunar valve insufficiency or stenosis can be addressed surgically without transplantation. In a subset of patients, the risks of valve repair or replacement are high, especially if multiple valves are involved, usually in the setting of single-ventricle physiology. Rarely, this may occur in patients with biventricular CHD. Primary heart transplantation should be considered on an individualized basis.
  - **Complex heterotaxy syndromes:** The vast majority of heterotaxy patients undergo surgical intervention: most undergo staged palliation to achieve a single-ventricle circulation, while a minority undergo biventricular repair. A small subset of patients with heterotaxy syndromes with severe atrioventricular or semilunar valvar abnormalities along with associated total anomalous pulmonary venous return, may be considered for primary heart transplantation in select circumstances [8].
- **Refractory life-threatening arrhythmias:** this is seen in a minority of patients, most of whom have structurally normal hearts without CHD. These ventricular arrhythmias can not be controlled by automatic implantable cardioverter defibrillator devices, medications, or ablation techniques. Transplantation may be offered as an option of last resort.

A recent analysis of 307 pediatric heart transplant patients from our institution transplanted over a 24 year period [9] revealed that CHD was present in 57% of patients, while cardiomyopathy was present in 39% and retransplantation in 4%. Among those with CHD, biventricular anomalies were present in 20% (35 of 174 patients). In our series, TGA was the most common diagnosis in patients with biventricular CHD, accounting for 31% of patients (11 of 35 patients). Aortic stenosis was present in 5 patients, atrioventricular canal defects in 4 patients, tetralogy of Fallot in 3 patients, and ventricular septal defects in 3 patients. Other anomalies were present in an additional 9 patients.

The largest cohort of patients with biventricular CHD undergoing heart transplantation comes from Lamour and coworkers [10] who merged the Pediatric Heart Transplant Study database (pediatric patients, 1993-2002, n=367) and the Cardiac Transplant Registry Database (adult patients, 1990-2002, n=121). They identified 488 adult and pediatric patients with univentricular and biventricular CHD who underwent cardiac transplantation at six months of life or later. A total of 366 patients younger than 6 months of age were excluded since 80% of them underwent primary heart transplantation for CHD; the rest underwent transplantation less than six months after their last surgery. Those two subsets were thought to represent patients whose results would not be generalizable to the older CHD patients. Most of the excluded patients had univentricular CHD, although some had biventricular CHD. There were 176 patients (36%) with univentricular lesions among the remaining 488 patients. This left 312 patients with biventricular CHD (64%), whose various underlying lesions are shown in Table 1. While this study did not specifically stratify patients based on biventricular versus univentricular CHD, a number of comments can be made: Of the patients with biventricular CHD, TGA, either D- or L-TGA, accounted for the biggest subset of biventricular CHD patients (97 patients, 31%). This was due to systemic ventricular failure, often a systemic right ventricle. The second largest subgroup represented those with either right or left ventricular outflow tract anomalies (87 patients, 28%). Most right ventricular outflow tract lesions had tetralogy of Fallot. Patients with septal defects (atrial, ventricular, and atrioventricular septal defects) represented the third largest cohort (75 patients, 24%). 

The group at Columbia University analyzed 106 patients with complex congenital heart disease, both univentricular and biventricular, who underwent heart transplantation [11]. Unlike the earlier study [10], patients younger than six months of age were not excluded. As a result, single ventricle lesions predominated, and accounted for 58.5% of all patients. Biventricular CHD was present in 44 patients, with transposition representing the biggest cohort (D-TGA in 10, L-TGA in 6), followed by right ventricular outflow tract lesions (tetralogy of Fallot in 9 and PAIVS in 8), Ebstein’s anomaly in 3, and other diagnoses in 13. Some of the PAIVS patients were single ventricle patients. The study did not differentiate between univentricular and biventricular CHD with respect to outcomes analysis. 

As a result of the above studies, patients with biventricular CHD undergoing heart transplantation belong to the following three patient substrates:

- ***Progressive systemic right ventricular failure:*** This occurs in the setting of unrepaired or physiologically-repaired congenitally-corrected TGA or following atrial switch procedures for D-TGA [12]. It represents the most common indication for heart transplantation in older patients with biventricular CHD [2].
- ***Progressive systemic left ventricular failure:*** A number of these patients may have septal defects (ventricular, atrial, and atrioventricular septal defects). Patients with coronary complications following the arterial switch procedure for D-TGA also fall in this category.
- ***Progressive right ventricular failure:*** This occurs primarily in patients with tetralogy of Fallot or double outlet right ventricle following surgical repair, where the systemic ventricle is a left ventricle. Severe Ebstein’s anomaly also falls into this category.

## CONTRAINDICATIONS

There are a number of contraindications to heart transplantation, which are covered elsewhere in this issue. The following list represents contraindications to transplantation, which applies to patients with biventricular CHD as well as any other patients undergoing consideration for heart transplantation:

- **Multiorgan dysfunction or failure:** severe hepatic or renal dysfunction is a relative contraindication since low cardiac output may be the reason behind the liver or kidney disease. Patients with either liver or kidney disease are at increased risk for early mortality following heart transplantation [3]. Often, renal dysfunction improves following heart transplantation. This needs to be balanced against the fact that calcineurin inhibitors necessary for immunosuppression are associated with renal insufficiency. Combined pediatric heart-kidney and heart-liver transplant procedures have been reported but are reserved for very select circumstances.
- **Elevated irreversible pulmonary vascular resistance** (PVR): A determination of PVR is essential for listing for heart transplantation, and is especially applicable to patients with biventricular CHD. In the past, a PVR > 4 Wood units*m^2^ without response to pulmonary vasodilators was considered a contraindication for heart transplantation [13]. With advances in postoperative care, and with development of treatment algorithms for patients with pulmonary hypertension, these guidelines have been relaxed. In the most recent decade, it is now acceptable to transplant patients with a PVR below 6 Wood units*m^2^ and a transpulmonary gradient below 15 mm Hg [14]. Patients with a PVR between 6-7 Wood units*m^2^ may still be considered for transplantation albeit at increased risk. Other centers have used a cutoff of 5 Wood units*m2 and a transpulmonary gradient below 12 mm Hg [15]. If PVR is >7 Wood units*m^2^, then physiologic testing with pulmonary vasodilators, such as inhaled nitric oxide, 100% oxygen, prostaglandins, or adenosine, should be performed. If the PVR drops below 7 Wood units*m^2^, then transplantation may be offered, although the perioperative mortality and morbidity will be increased due to pulmonary hypertension. Careful donor selection is important to achieve an acceptable outcome in these high-risk patients. Some have advocated the use of oversized male donors in order to improve perioperative outcomes. Pulmonary vasodilators, including inhaled nitric oxide and milrinone, should be used liberally in the postoperative period. If the PVR does not fall with physiologic testing during the pre-transplant evaluation, then patients should be placed on chronic pulmonary vasodilators such as intravenous prostacyclin, oral sildenafil or bosentan, in combination with inotropic support in order to decrease the left ventricular end diastolic pressure. Repeat determination of the PVR should be performed every 3-6 months. In some patients, pulmonary vascular remodeling may render them candidates for heart transplantation. In others, implantation of a ventricular assist device can result in a decrease in the PVR, thought to be due to unloading of the left ventricle, resulting in a decrease in the left atrial pressure [16-18]. Another option is combined heart-lung transplantation, although the long-term outcomes with heart-lung transplantation are significantly worse compared with isolated heart transplantation. An alternative is heterotopic heart transplantation, where the transplanted heart acts as a biological ventricular assist device.
- **Incurable malignancy:** Active malignancy in the recipient is a contraindication for heart transplantation. The dilemma arises when there has been remission for a period of time. Listing for heart transplantation in this setting should be individualized, taking into account the expected cancer-free survival of the patient.
- **Uncontrolled infection: **Heart transplantation should be deferred until the infection has been controlled. An exception is driveline infections in the setting of ventricular assist devices, which can be controlled but not eliminated. Heart transplantation will treat the underlying cardiac dysfunction as well as the infection by removal of the offending hardware.
- **Human immunodeficiency virus:** Most centers still consider this an absolute contraindication for heart transplantation, although advances in retroviral therapy have led some to offer heart transplantation in this setting [19].
- **Significant psychosocial problems:** The success of organ transplantation depends on the presence of a supportive family to keep regular follow-up appointments and to comply with various treatment protocols. Medication non-compliance is a significant problem especially in the teenage transplant recipient who is doing well. This represents a leading cause of late graft failure. Developmental delay is commonly seen in patients with CHD considered for heart transplantation [14]; this alone should not be a contraindication for heart transplan-tation. We and others have transplanted select patients with Down syndrome. Consideration for transplantation should be individualized, taking into consideration the overall resources available to the patient and the family.

It should be emphasized that there are very few recipient anatomic factors that contraindicate heart transplantation. Significant recipient pulmonary artery hypoplasia or pulmonary vein stenosis result in increased perioperative mortality and morbidity; transplantation in those settings should be individualized. Various recipient anatomic considerations are discussed separately below. 

## DONOR EVALUATION

Our donor evaluation and harvesting technique has been standardized, and is described in detail elsewhere [20]. We avoid donors who are on high doses of multiple inotropes or vasoactive agents, unless absolutely necessary. The size discrepancy between donors and recipients varies with recipient age: for neonates, we consider donors who are as much as three times the weight of the recipient, whereas for older children, we prefer donors who are within 20% of the recipient weight. If the weight discrepancy is significant, delayed sternal closure should be performed following diuresis and resolution of allograft myocardial edema. In addition, the leftward aspect of the pericardium often needs to be resected and the left pleural space opened in order to allow the cardiac apex to rest comfortably in the recipient’s mediastinal space.

It is important to obtain as much donor tissue as is feasible, in order to aid in the surgical reconstruction at the time of implantation. Using recipient anatomy as a guide, this advance planning simplifies the implant procedure. We prefer to harvest the entire length of the superior vena cava, the left innominate vein, and the entire aortic arch including the proximal portion of the descending thoracic aorta. If the lungs are not harvested, then we prefer to harvest the branch pulmonary arteries from hilum to hilum. In addition, donor pericardium should be harvested, as it can serve as additional tissue for reconstruction. We do not trim the donor organ until the time of implantation.

## SURGICAL TECHNIQUE

The technical challenges in performing the cardiac transplant procedure have been overcome, even for the most anomalous recipient anatomic lesions. In the setting of experienced surgical transplant centers, outcomes of heart transplantation for CHD approach but remain inferior to those for cardiomyopathy, due to recipient factors and the increased complexity of the surgical implant procedure.

Care should be taken during redo sternotomy to avoid catastrophic mediastinal hemorrhage. It is important to carefully plan for the operation, including review of prior operative reports and imaging studies. We routinely obtain preoperative chest CT scans with intravenous contrast in order to identify retrosternal structures and locations of major vessels. The groin vessels often need to be exposed. Following recipient cardiectomy, it is important to achieve surgical hemostasis prior to allograft implantation.

Residual recipient anatomic lesions that are not addressed by recipient cardiectomy should be addressed and repaired. Failure to address residual lesions is one reason for the higher mortality seen in earlier eras for patients transplanted for CHD [21]. In most univentricular lesions, this will require reconstruction of the pulmonary arteries, typically with patch material. This is not often needed in patients with biventricular CHD. 

Most cyanotic CHD patients who have undergone prior surgery will have aortopulmonary collaterals, which can result in a large amount of pulmonary venous return entering the left atrium. In addition to obscuring the surgical field, this increased return also rewarms the allograft during implantation. This can be addressed by using a left ventricular vent, decreasing the flow rate on cardiopulmonary bypass (CPB), and cooling to a lower temperature. 

There are three options for implantation of the allograft in the recipient:

- **Bicaval technique:** This is the most common method used for implantation, as studies have shown a lower associated risk of tricuspid insufficiency, improved right atrial transport function and less atrial arrhythmias. Some patients may develop stenosis at the superior vena cava (SVC) anastomosis, which is amenable to balloon dilation and stent implantation. This technique also allows for greater flexibility in anatomic reconstruction of the underlying CHD anomaly.
- **Biatrial technique:** In recipients smaller than 1 year of age, the SVC may be small and fragile, and the risk of a postoperative anastomotic stricture may be high. In that setting, a biatrial technique is advocated, whereby the donor right atrium is anastomosed to the recipient right atrium, instead of performing individual caval anastomoses.
- **Individual pulmonary vein technique:** There is little data to support performing isolated right and left pulmonary venous anastomoses, although this has been advocated by some in adult heart transplantation, in order to decrease the incidence of left atrial thrombus formation and resultant thromboembolism [22]. In the pediatric population, individual pulmonary venous anastomoses are more difficult, especially in recipients with dilated left atria where there is wide separation of the recipient pulmonary venous ostia. The technique may also result in a higher incidence of atrial arrhythmias and pulmonary vein stenosis.

Various anatomic subtypes of biventricular CHD require variations in the implant technique. These are discussed below.

### Bilateral superior vena cavae

**Fig. (1):**
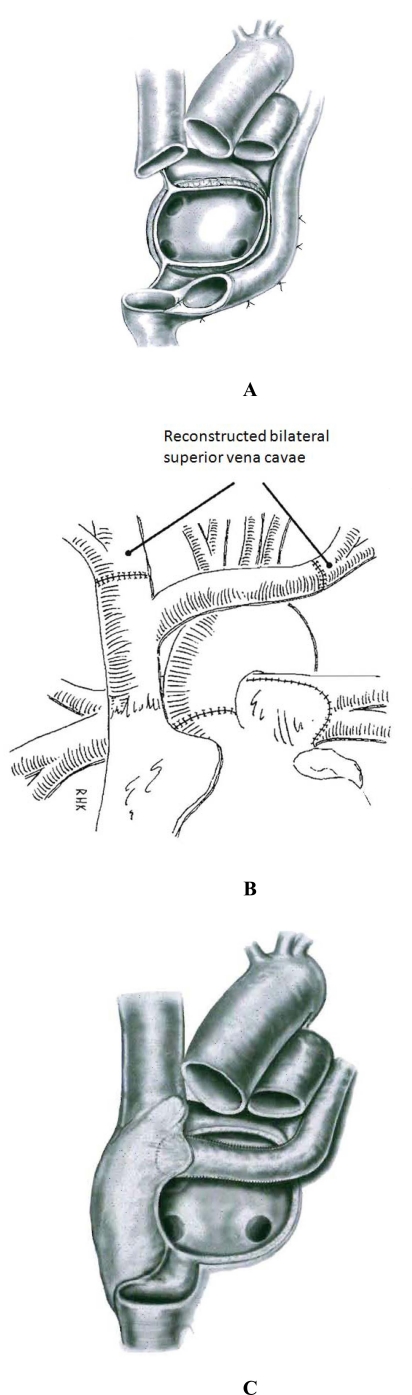


A left SVC, when present, most commonly drains into the right atrium through a dilated coronary sinus. We prefer to alter the recipient cardiectomy so as to keep the coronary sinus and inferior vena cava together *en bloc*, which is sutured to the donor inferior vena cava (Fig. **1A**). Alternatively, the left SVC may be anastomosed to the donor innominate vein, although this can result in stenosis or bleeding due to the fragile nature of the innominate vein, especially in smaller patients (Fig. **1B**). 

In rare instances, a left SVC may drain directly into the roof of the left atrium, known as a Raghib association. In this situation, the left SVC is repaired either by an anastomosis to the left innominate vein (Fig. **1B**) or by construction of an intraatrial baffle from the roof of the left atrium to the right atrium (Fig. **1C**). The latter technique increases CPB time and myocardial ischemia time, and may negatively impact early perioperative outcomes.

### Congenitally-Corrected Transposition of the Great Arteries

Patients with CCTGA represent a large percentage of patients with biventricular CHD undergoing heart transplantation. In the most common form of this lesion, {S,L,L} CCTGA, the right atrium is right-sided and receives systemic venous return. The blood drains via the mitral valve into the anatomic left ventricle, which is right-sided and represents the pulmonary ventricle. The left atrium is left-sided and receives pulmonary venous return. The blood drains via the tricuspid valve into the anatomic right ventricle, which is left-sided and represents the systemic ventricle. The aorta is usually to the left and slightly anterior to the main pulmonary artery. Associated abnormalities include the presence of a ventricular septal defect, pulmonary stenosis, and Ebsteinoid malformation of the tricuspid valve. A less common form is CCTGA with situs inversus, known as {I,D,D} CCTGA.

In older eras, cardiac surgical palliation was in the form of simpler procedures that maintained the morphological right ventricle as the systemic ventricle. With time, this systemic right ventricle failed, resulting in systemic right ventricular dilatation and systemic tricuspid regurgitation. Over the past decade, the double switch procedure has been performed more liberally, which is a combination of an atrial and an arterial switch procedure. This results in a systemic left ventricle, and is expected to improve long-term outcomes.

At the time of transplantation, the recipient’s ascending aorta is transected quite superiorly, where the aorta is closer to the midline. The main pulmonary artery is transected at the pulmonary artery bifurcation, and the incision is extended into the proximal origin of the left pulmonary artery. The rightward aspect of the pulmonary arteriotomy is closed, which moves the pulmonary artery bifurcation leftward. This avoids kinking of the pulmonary arteries by the midline ascending aorta.

**Fig. (2):**
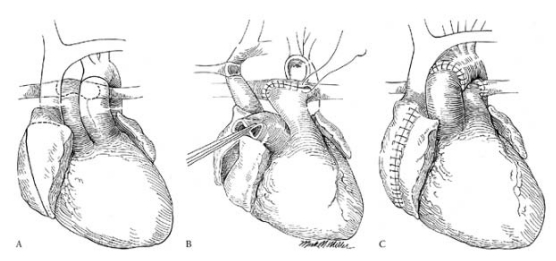


During implantation, the pulmonary artery anastomosis is done before the aortic anastomosis, in order to allow a clear and unobstructed view of the surgical field. The donor ascending aorta is kept long and anastomosed to the distal as pect of the recipient ascending aorta. This moves the position of the ascending aorta rightward, corresponding to the leftward move in the main pulmonary artery anastomosis done earlier (Fig. **2**).

### Situs inversus

**Fig. (3):**
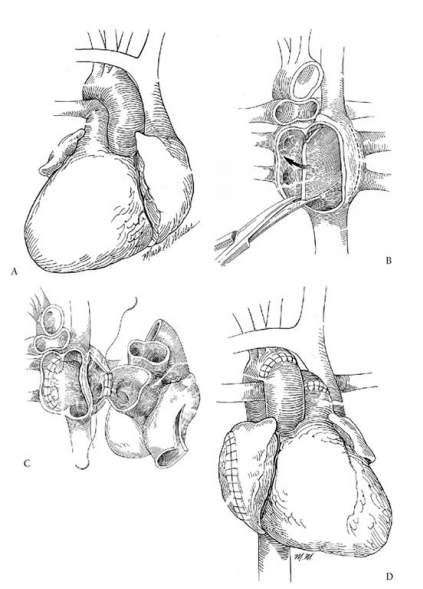


Situs inversus occurs at a rate of 2/10,000 population. Patients may have severe CHD associated with single ventricle physiology, or may have isolated cardiomyopathy as in biventricular CHD. In situs inversus, the right atrium is left-sided, along with the SVC and inferior vena cava. The left atrium and pulmonary veins are right-sided. Dextrocardia is often present, with the cardiac apex pointing to the right. The aorta is to the left of the main pulmonary artery (Fig. **3A**).

The main surgical challenge lies in routing of the systemic venous return in the transplanted heart. During cardiectomy, the atrial septum should not be resected, as it will be used to reroute pulmonary venous drainage. The aorta and main pulmonary artery are transected distally. The recipient atrial septum is mobilized by dividing it superiorly and inferiorly for extra mobility. This is then anastomosed to the left atrial free wall, anterior to the right pulmonary veins. A separate atriotomy is made anterior to the left pulmonary veins, which will serve as the recipient neo-left atrium, which is now left-sided, corresponding to the donor heart. The leftward aspect of the right atrial cuff is closed, which moves the right atrial cuff rightward. The right atrial anastomosis is performed after the left atrial anastomosis. The pulmonary artery anastomosis is performed next, using a similar technique as described above for CCTGA, whereby an incision is made in the left pulmonary artery, which moves the pulmonary artery anastomosis leftward (Fig. **3**).

### Heterotaxy

Heterotaxy syndromes (also known as right or left atrial isomerism) often accompany situs inversus lesions. There are abnormalities of systemic and pulmonary venous return. The majority of heterotaxy patients fall in the univentricular CHD category. The recipient operation combines elements of CCTGA and situs inversus. Our technique has been presented elsewhere [23], and is beyond the scope of this manuscript.

### Dextrocardia

Often, dextrocardia is associated with situs inversus, which was addressed previously. If dextrocardia is present with a normal situs solitus, then heart transplantation is challenging, as this may lead to a rightward rotation of the cardiac apex, which may distort the tricuspid valve and the interventricular septum. If the pericardial space is dilated, this is not a major concern. If the pericardial space is not enlarged, then the left pericardium should be excised in order to allow the cardiac allograft to be positioned normally in the left chest.

## SURVIVAL

Overall survival following heart transplantation for all types of CHD (univentricular and biventricular) is lower compared to patients with cardiomyopathy, based on large registry studies. This is true for both pediatric and adult heart transplant recipients, and is primarily related to increased perioperative mortality. After excluding the perioperative period, survival for patients with CHD is similar to other diagnoses [14].

Based on the most recent analysis of the International Society for Heart and Lung Transplantation Registry, CHD is a risk factor for one-year survival [3]. The highest risk factor for mortality at 1 year following transplantation is the presence of CHD in a neonate on ECMO, with a relative risk of 2.86. Non-neonates with CHD still accounted for a relative risk for 1-year mortality of 2.0. This increased risk for mortality in CHD recipients persisted in adolescents 11-17 years of age, with a relative risk for 1-year mortality of 1.86 [3]. Our center’s results mimic those from the ISHLT Registry [9]. Our overall survival following heart transplantation is 84%, 77%, and 72% at 1, 5, and 10 years, respectively. Patients with CHD had somewhat lower survival at 80%, 73%, and 66% at 1, 5, and 10 years, respectively. Single ventricle patients following palliation had the worst outcomes, at 70%, 58% and 50% at 1, 5, and 10 years, respectively. It should be noted, however, that a number of single-center studies from earlier eras have reported similar survival for patients with CHD and cardiomyopathy, although with smaller sample sizes and limited power [24-26]. This is likely not applicable to the current era due to increased patient complexity for those with CHD: borderline patients are being transplanted who previously would not have been considered for transplantation, and the cohort of HLHS patients undergoing primary transplantation in earlier eras has virtually disappeared.

Decreased survival for patients with CHD undergoing heart transplantation is likely due to several reasons:

- **Reoperation:** most cardiomyopathy patients have not had previous surgery, while most CHD patients have had prior surgical procedures. This increases the risk of bleeding and the technical complexity of the operation. Of the 488 patients with univentricular and biventricular CHD patients older than six months of age that were analyzed by Lamour and coworkers, 93% had undergone previous cardiac surgical procedures [10]. It is difficult to discern what percentage of the biventricular CHD patients had prior surgery, but presumably the result would be quite similar to the overall cohort. The number of previous sternotomies increased the ischemic time due to the greater need for dissection on cardiopulmonary bypass: those with 1-2 pervious sternotomies had an average ischemic time of 228 minutes, while those with >3 previous sternotomies had an average ischemic time of 242 minutes. The predicted probability of death increased in older recipients with older donors and longer ischemic times. Longer ischemic time was a risk factor for survival (p=0.02).
- **Reconstruction:** most patients with CHD will need to undergo reconstruction of the pulmonary arteries or aorta, or need reconstructive procedures on their systemic or pulmonary venous drainage pathways. This is especially true for heterotaxy or situs inversus patients, some of whom may have biventricular CHD, although most will have single-ventricle anatomy. As a result, the reconstructive procedures that need to be performed along with the heart transplant procedure add to the technical complexity of the procedure, prolong cardiopulmonary bypass time, and increase short-term morbidity and mortality.
- **Antibody sensitization:** Previous surgical procedures with exposure to blood products, along with prior use of homograft tissue, increase the amount of preformed antibodies measured by the panel reactive antibody (PRA) test. A PRA > 10% typically limits the donor pool to the local transplant region, increasing waiting list mortality. Alternatively, patients may be transplanted across a positive cross-match, which often increases post-transplant mortality and morbidity due to increased episodes of rejection (cellular or humoral) and the need for augmented immunosuppression.
- **Elevated pulmonary vascular resistance:** Some CHD patients have an elevated PVR, which often requires prolonged postoperative sedation and neuromuscular blockade. This increased postoperative pulmonary vascular lability contributes to worse perioperative outcomes.

Other factors contributing to operative mortality in patients with CHD include the clinical status of the recipient at the time of transplantation, the mismatch between donor and recipient sizes, the presence of aortopulmonary collateral vessels, the degree of pulmonary and systemic venous return anomalies, and the malalignment of the great vessels in patients with CHD [14, 27].

Graft failure is the most common cause of early mortality following heart transplantation, especially in neonates. Supportive strategies include maximal medical management, especially aimed at treating right ventricular dysfunction, including nitric oxide and prostacyclin, neuromuscular blockade, and ventilation with 100% FiO2. 

Mechanical circulatory support, if needed, is extracorporeal membrane oxygenation (ECMO) in neonates and young infants. Older children may benefit from temporary centrifugal circuits such as the CentriMag ventricular assist device.

The course of mechanical circulatory support is usually one week or less, in order to allow improvement and recovery of cardiac allograft function. 

A number of risk factors associated with early phase mortality and constant phase mortality have been identified in CHD patients with biventricular and univentricular lesions [10]. Those are listed in Table 2. While risk factors for CHD patients with only biventricular anomalies were not specifically studied, a number of inferences may be made. First, longer ischemic times are commonly present in patients with biventricular CHD, due to the need to perform redo sternotomy along with further dissection on cardiopulmonary bypass prior to recipient cardiectomy. This would result in increased mortality, as survival was generally worse with prolonged ischemic times, p=0.02. Second, older recipient age is also commonly present in patients with biventricular CHD, further increasing the relative risk for heart transplantation. Overall survival also varies depending on the underlying CHD anomaly. For example, patients with TGA had the highest Kaplan-Meier survival, at 88%, compared to those with atrioventricular canal defects, which fared the worst, at 62% survival, p=0.02. 

In a study from Columbia University on heart transplantation for complex congenital heart disease, there was a trend for various anatomic subgroups to have different odds ratio for overall mortality, although this was not statistically significant due to small sample sizes and variable periods of follow-up [11]. Patients with tetralogy of Fallot had an odds ratio for mortality of 2.0, transposition of the great arteries had an odds ratio of 1.6, pulmonary atresia had an odds ratio of 1.4, while pulmonary stenosis had an odds ratio of 1.2. In that study, patients who underwent any pulmonary artery reconstruction had inferior Kaplan-Meier survival curves compared to patients who did not require pulmonary artery reconstruction, p=0.053. Regression analysis of survival revealed that any pulmonary artery reconstruction carried an odds ratio of 3.3, while transplantation in a neonate carried an odds ratio of 5.2; male gender was protective with an odds ratio of 0.5, and a more recent year of transplantation was mildly protective with an odds ratio of 0.9.

**Fig. (4):**
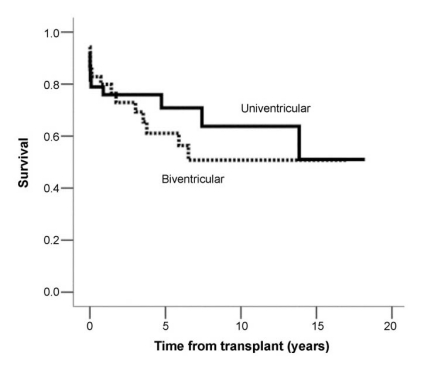


A recent analysis by the Great Ormond Street Hospital for Children of 73 patients with CHD treated since 1988 revealed similar survival for patients with univentricular and biventricular CHD anomalies [28]. One-year survival for univentricular physiology was 75% vs 78% for biventricular physiology, p=NS. At one-month post-transplant, a total of 8 of 38 univentricular patients and 5 of 35 biventricular patients had died, p=NS. See Fig. **4**. This result, however, may be due to the small sample size, as this has not been reproduced in most studies.

## POST-TRANSPLANT SEQUELAE

A number of post-transplant complications have been recognized following heart transplantation, applicable to all recipient subgroups. These have been extensively reviewed elsewhere [29], and are beyond the scope of this manuscript. Issues specific to patients with biventricular CHD are addressed below.

### Bleeding

The need for redo sternotomy in the setting of palliated or repaired biventricular CHD increases the incidence of postoperative bleeding. Since aprotinin is no longer available, other antifibrinolytic agents such as tranexamic acid or epsilon aminocaproic acid have been used, although their efficacy has not been demonstrated. 

### Acute Rejection

Although neonates and young infants have higher perioperative mortality, they experience lesser rates of acute rejection and often can be managed with decreased levels of immunosuppression. This is due to a naïve and immature immune system, which also allows for ABO-incompatible heart transplantation. Patients with biventricular CHD often have elevated panel reactive antibodies, which increase the incidence of acute cellular rejection and antibody-mediated rejection. This is thought to be a major factor influencing the lower survival following heart transplantation in this group compared to patients with cardiomyopathy.

### Post-Transplant Lymphoproliferative Disease

This complication occurs in 5-10% of patients postoperatively. Specific studies in patients with biventricular CHD have not been performed.

### Cardiac Allograft Vasculopathy

When compared to cardiomyopathy, CHD increases the incidence of CAV within five years following heart transplantation, with a relative risk of 1.36 based on the most recent ISHLT Registry analysis, p=0.08 [3]. This increased incidence of chronic rejection likely stems from increased preformed antibodies as well as the need for greater blood products during redo sternotomy procedures. The prior use of homograft tissue, when present, also increases the incidence of preformed antibodies and likely contributes to a greater incidence of acute and chronic rejection.

## CONCLUSIONS

Patients with biventricular CHD represent a heterogeneous group of patients with varied anatomic substrates, prior surgeries, age, and clinical status. With increasing experience, results for heart transplantation in this subgroup continue to improve, although are still inferior to transplantation for cardiomyopathy. It is hoped that further refinements in patient selection, techniques, and postoperative management will result in continued improvements in outcomes of heart transplantation for patients with biventricular congenital heart disease. 

## Figures and Tables

**Table 1.** The Distribution of Various Diagnoses Among Pediatric and Adult Patients with Biventricular CHD > 6 Months of Age who Underwent Heart Transplantation Between 1990-2002. Adapted from (Lamour 2009).

	Number	Percentage
D-Transposition of the great arteries	58	19%
Right ventricular outflow tract lesions	49	16%
Ventricular and/or atrial septal defects	38	12%
Left ventricular outflow tract lesions	38	12%
L-Transposition of the great arteries	39	13%
Complete atrioventricular canal defect	37	12%
Other biventricular CHD lesions	53	17%
TOTAL	312	100%

**Table 2.** Risk Factors Associated with Early Phase and Constant Phase Mortality in All Pediatric and Adult Patients with Univentricular and Biventricular CHD > 6 Months of Age who Underwent Heart Transplantation Between 1990-2002. From (Lamour 2009).

Variable	Relative Risk	p Value
**Early phase**		
Previous Fontan operation	8.6	0.003
Higher pre-transplant mean RAP (only in those with a previous Fontan)	2.4	<0.0001
Longer ischemic time	1.6	0.002
Older recipient age	1.5	0.02
Interaction of donor age and ischemic time	1.4	0.0007
		
**Constant phase**		
Previous classic Glenn operation	3.1	0.01
CMV+ donor, CMV- recipient	2.8	0.001
Higher systolic transpulmonary gradient	2.0	0.01
Younger recipient age	1.8	0.0001

## ABBREVIATIONS

CCTGA: = Congenitally-corrected transposition of the great arteries
CHD: = Congenital heart disease
CPB: = Cardiopulmonary bypass
ECMO: = Extracorporeal membrane oxygenation
HLHS: = Hypoplastic left heart syndrome
PAIVS: = Pulmonary atresia with intact ventricular septum
PRA: = Panel reactive antibody
PTLD: = Post-transplant lymphoproliferative disease
PVR: = Pulmonary vascular resistance
RVDCC: = Right ventricle dependent coronary circulation
SVC: = Superior vena cava
TGA: = Transposition of the great arteries
